# Adaptation to bipolar disorder and perceived risk to children: a survey of parents with bipolar disorder

**DOI:** 10.1186/1471-244X-13-327

**Published:** 2013-12-02

**Authors:** Holly L Peay, Donald L Rosenstein, Barbara B Biesecker

**Affiliations:** 1 Social and Behavioral Research Branch, National Human Genome Research Institute NHGRI, Building 31 Room B1B36 31 Center Drive MSC 2073, Bethesda, MD 20892, USA; 2 Department of Psychiatry and Comprehensive Cancer Support Program, University of North Carolina at Chapel Hill, Physicians Office Building, Room 3134, Chapel Hill, NC 27599, USA

**Keywords:** Bipolar disorder, Adaptation, Quality of life, Genetic, Risk perception

## Abstract

**Background:**

Bipolar disorder (BPD) is a common condition associated with significant morbidity and reduced quality of life. In addition to challenges caused by their mood symptoms, parents affected with BPD harbor concerns about the mental health of their children. Among adult parents who perceive themselves to have BPD, this study aims to examine participants’ coping methods; identify predictors of adaptation; assess parental perceptions of risks for mood disorders among their children; and describe the relationships among illness appraisals, coping, adaptation to one’s own illness, and perceived risk to one’s children.

**Methods:**

Parents who self-identified as having BPD completed a web-based survey that assessed dispositional optimism, coping, perceived illness severity, perceived etiology of BPD, perceived risk to offspring, and adaptation to BPD. Participants had at least one unaffected child who was 30 years of age or below.

**Results:**

266 parents were included in the analysis. 87% of parents endorsed a “somewhat greater” or “much greater” risk for mood disorders in one’s child(ren) than someone without a family history. Endorsing a genetic/familial etiology to BPD was positively correlated with perceived risk for mood disorders in children (*r*_s_ = .3, p < 0.01) and active coping with BDP (*r* = .2, p < 0.01). Increased active coping (β = 0.4, p < 0.001) and dispositional optimism (β = 0.3, p < 0.001) were positively associated with better adaptation, while using denial coping was negatively associated with adaptation (β = −0.3, p < 0.001). The variables explained 55.2% of the variance in adaptation (F = 73.2, p < 0.001). Coping mediated the effect of perceived illness severity on adaptation.

**Conclusions:**

These data inform studies of interventions that extend beyond symptom management and aim to improve the psychological wellbeing of parents with BPD. Interventions targeted at illness perceptions and those aimed at enhancing coping should be studied for positive effects on adaptation. Parents with BPD may benefit from genetic counseling to promote active coping with their condition, and manage worry about perceived risk to their children.

## Background

Bipolar disorder (BPD) represents a group of common, disabling conditions of mood dysregulation. The improved ability to control overt illness symptoms through psychopharmacological and psychosocial treatments, as highlighted in the Systematic Treatment Enhancement Program for Bipolar Disorder (STEP-BD) study [1], has afforded new opportunities for people with mental illness. However, studies suggest that functional and psychological well-being of individuals with bipolar disorders is not consistently predicted by symptom severity [2,3]. As evidence for the management of BPD symptoms improves, it is important to broaden our understanding of factors that contribute to psychological well-being (subjective assessments of life quality, including emotional reactions and cognitive judgments), beyond overt symptoms and predictors of symptom exacerbation and remission.

A qualitative study of adults with BPD and their healthy siblings [4] reinforced the need to further explore the effects of BPD on the psychological well-being of affected individuals, at-risk children, and the family unit. Participants described distress around their perceived family vulnerability to mood disorders. Many described concerns about negative effects of BPD on their parenting abilities and the family, and concerns that these negative effects may increase the risk of mood disorders in offspring--perceptions supported by prior research [5,6]. Participants who perceived themselves to be better adapted to BPD seemed to better manage the effects of BPD on the family unit, and have lower concern about psychological outcomes in their children. Our qualitative study led to interest in evaluating, among parents with bipolar disorder, predictors of adaptation to BPD and whether there is a relationship among adaptation, perceived risk to children, and parental coping with risk to children.

### Well-being in bipolar disorder

Most studies of well-being in BPD measure quality of life (QoL). QoL is an assessment, typically performed at one time period, of multiple domains used to represent global well-being. QoL assessments are typically applied to those managing a disease or condition. Individuals with BPD have lower QoL than individuals in the general population [2,7,8]. QoL has been shown to be significantly positively correlated with adaptation, which is another component of psychological wellbeing; however, they have been found to be associated with different predictor variables [9]. Adaptation is a measurable, positive outcome of coping with the stress of a health condition and consists of restored self-esteem, existential well-being, social reengagement, and coping efficacy [9].

Well-established predictors of psychological well-being include coping and personality traits, which exerting their influence through independent and interactive roles [10]. Efforts to prevent or diminish threat, harm, and loss, or to reduce associated distress are described as coping strategies [10]. Little is known about coping with BPD *beyond* coping with symptoms; coping in BPD has been primarily evaluated as a predictor of symptom response rather than psychological wellbeing [11,12].

Existing studies of psychological well-being in BPD fall short in identifying patients’ perceptions of their abilities to manage BPD. For example, there is limited data on the appraisals that engage use of coping strategies and whether they are effective in enhancing adaptation [13]. Studying contributors to adaptation to BPD, including illness perceptions and coping, broadens our understanding of the inter-relationship of these concepts and can inform the design of intervention studies aimed at improving psychological wellbeing.

### Concerns about mood disorders in children

Stressors facing parents with BPD may include worries about risk of mood disorders in their children, and how to manage those risks [4]. A significant proportion of individuals affected with serious psychiatric disorders have concerns about their children’s risks for psychiatric disorders [14-16]. These concerns are warranted by the high heritability of BPD, estimated at 85% [17], and a 20%-30% estimated lifetime risk for a mood disorder in a first-degree relative of an individual with BPD [18,19]. Affected individuals tend to appreciate the etiological complexity, attributing illness causation to a range of genetic and environmental factors [4,20,21].

The negative impact of parental BPD on family functioning and increased risk to offspring [5,6], together with data showing that affected parents are aware of genetic and environmental avenues for increased risk to children [4], supports investigating the relationship between parental adaptation to their BPD and perceived risk to their children. Data about the factors that influence risk perception may inform genetic counseling and suggest whether interventions targeted at disorder adaptation may impact perception of risk to children.

Among adult parents who perceive themselves to have BPD the specific aims were to:

1. Examine participants’ coping methods and test bivariate relationships with coping, anticipating the importance of coping as a predictor of adaptation [10];

2. Identify predictors of adaptation to BPD;

3. Assess parental perceptions of risks for mood disorders in their children; and

4. Describe the relationships among illness appraisals, coping, adaptation to one’s own illness, and perceived risk to one’s children.

We hypothesized that higher adaptation to bipolar disorder would be predicted by dispositional optimism, lower perceived illness severity, and coping type.

## Methods

This cross-sectional survey was self-administered online. The survey was listed by mental health advocacy organizations (including National Alliance on Mental Illness, Bipolar World, Bipolar Significant Others, and Depression and Bipolar Support Alliance) and word-of-mouth recruiting. Adults who report as having 1) BPD and 2) at least one unaffected biological child aged 30 or younger were eligible to participate. Recruitment was limited to parents of children 30 years or younger because the average age at BPD onset is in the late 20s [18].

Our intent was to understand the perceptions of individuals who *identify themselves as having* BPD. Similar to the majority of cross-sectional surveys of disorder populations, especially online surveys, the accuracy of participant self-identification was assumed; i.e., the survey does not include a measure to evaluate mental health status. This approach is consistent with on-line surveys of many populations and offers the opportunity for greater understanding of the lived experiences of patients in a feasible manner.

This study was approved by the National Human Genome Research Institute’s Institutional Review Board. Informed consent was presumed by the participants’ willingness to complete the online survey.

### Measures

Data include respondents’ age, age at diagnosis, sex, ethnicity, marital status, state of residence, number of child(ren), and age of child(ren). For those with more than one child, we collected data about birth order and sex of the child they worried about the most, and included an open-ended question about why this child was associated with the most worry. The study was framed by the Transactional Theory of Stress and Coping [13].

### Predictor variables

#### Illness characteristics and perceptions

The survey included one question each about whether the participant perceived him/herself as currently manic or currently depressed. The response options were “yes”, “no”, or “uncertain”; results were dichotomized to yes or no/uncertain. The survey also included a query about the participant’s degree of confidence that BPD is the diagnosis that best explains his/her symptoms, scored on a 1–5 scale of “not at all” to “very much.”

#### Perceived illness severity (Brief Illness Perceptions Questionnaire)

The Brief IPQ [22] measured self-assessed illness severity. The measure has previously been used in populations of individuals with mental illness [23,24]. The 8 items, on a scale of 0–9 anchored with extremes (e.g., “No effect at all” to “Severely affects my life”), were summed and higher scores indicate increased severity. In this sample, Cronbach’s alpha was 0.7.

#### Perceived etiology of BPD

Based on a past qualitative study and clinical experience, we developed a new measure of people’s perceptions about “how bipolar disorder happens in families.” Principle components analysis (PCA) on the 5-item Perceived Etiology Measure revealed a two-component solution that explained 77% of the variance. Factor 1 included genetic and familial items and factor 2, attributes and environment. The items from each factor were averaged, with higher scores indicating increased endorsement. Cronbach’s alpha was 0.7 for factor 1 and 0.8 for factor 2. See Additional file 1.

#### Dispositional optimism (Life Orientation Test, LOT)

The LOT [25] was used to measure participants’ dispositional optimism. We were unable to identify a study that used this measure in a population with bipolar disorder; however, it is frequently evaluated as a moderator of psychological impairment in a target population (e.g., Thomas and colleagues, 2011) [26]. The 8 scored items (scale of 0–4, “strongly disagree” to “strongly agree”) were summed, with higher scores indicating greater optimism. Cronbach’s alpha was 0.9.

#### Coping with BPD

We used the 28-item Brief COPE [27] to assess coping with BPD. This measure has previously been used in a population with mental illness [28].

PCA identified the relevant coping domains in this population: a two-component solution using 16 items that explained 48% of the variance. Component 1, “active/social support coping,” loaded to 11 active coping/social support items and had a Cronbach’s alpha of 0.9. Component 2, “self-blame/denial coping,” loaded to 5 self-blame/denial items and had a Cronbach’s alpha of 0.7. For each factor, the items (on a 1–4 scale, “I usually don’t do this at all” to “I usually do this a lot”) were summed and averaged, with higher scores indicating increased use of the coping type.

### Outcome variables

#### Psychological adaptation scale (PAS)

Adaptation to bipolar disorder was measured using the PAS, comprising self-esteem, social integration, spiritual/existential meaning, and coping efficacy domains [9]. The 20 items (on a 1–5 scale, “not at all” to “very much”) were averaged, with higher scores indicating increased adaptation. This measure has not been used in a population with mental illness. In other populations, alpha scores of reliability have ranged from 0.83 to 0.97 [9] in previous studies. Cronbach’s alpha in this sample was 0.9.

#### Perceived risk to children

Perceived risk was assessed with the following item: “Compared to a child who does not have anyone in his/her family with a mood disorder, in my opinion MY child has a _________ chance to have a mood disorder,” with five response options ranging from ‘much smaller’ to ‘much greater’. Participants with more than one child were prompted to answer based on the child they worried about the most.

### Statistical analysis

Data were analyzed using the statistical software SPSS Statistics 17.0. Age of participant, age of child, time since diagnosis, number of children, confidence in diagnosis and self-report of current mania and current depression were defined as potential confounders. Bivariate associations between predictor variables, confounders, adaptation and perceived risk to children were examined using Pearson’s and Spearman’s correlations, respectively. Linear regression was used to assess the relationship between predictor variables and adaptation, controlling for confounders. We entered into the regression analysis all predictor variables with p < 0.25, then removed one variable at a time until only those with p values of <0.05 remained. We then added one potential confounder at a time and include any time the β if a predictor variable changes by more than 10%. To evaluate a post-hoc hypothesis that coping mediated the effect of illness perceptions on adaptation, we tested for mediation effects by using a series of regression analyses, as described by Vos et al. [29].

## Results

### Sample population

Two hundred and sixty-six parents with BPD completed the online survey. Table 1 shows the participant characteristics; the majority being Caucasian, female, married, and with more than one child. Participants resided in 43 states from across the United States. Participants’ ages were normally distributed. We did not collect any additional demographic data in this sample.

**Table 1:** Characteristics of study population

**Variable**		**N (%)**
Sex	Male	32 (16.3%)
	Female	164 (83.7%)
Race (choose all that apply)	White	181 (68.0%)
	Black/African-American	9 (3.4%)
	Native American or Alaskan Native	4 (1.5%)
	Asian	1 (0.4%)
	Hispanic or Latino	9 (3.4%)
	Other	4 (1.5%)
	No Race Chosen	68 (25.6%)
Age	18-25	12 (6.1%)
	26–35	44 (22.4%)
	36–45	73 (37.1%)
	46–55	60 (30.5%)
	56–65	8 (4.0%)
	Over 65	0
Marital status	Married	117 (59.7%)
	Separated or Divorced	56 (28.6%)
	Never Married	22 (11.2%)
	Widowed	1 (0.5%)
Time since diagnosis	Less than 1 year	29 (10.9%)
	1–5 years ago	88 (33.1%)
	6–10 years ago	67 (25.2%)
	11–15 years ago	44 (16.5%)
	16-20 years ago	23 (8.6%)
	More than 20 years ago	15 (5.6%)
Number of States represented		43

### Descriptive results

Table 2 shows means and standard deviations for the predictor and outcome variables.

**Table 2:** Means and standard deviation of key variables

**Predictor variable**	**Mean (SD)**
Dispositional optimism	13.5 (6.4)
Illness severity	46.0 (9.6)
Perceived etiology measure: genetic/familial component	4.2 (0.8)
Perceived etiology measure: attributes	2.8 (1.0)
Active/social coping	2.9 (0.7)
Self-Blame/denial coping	2.3 (0.7)
Adaptation	2.6 (1.0)
Perceived risk to child	4.2 (0.9)

The Perceived Etiology of BPD measure showed a significantly higher endorsement of genetic/familial etiology than attribute/environment, *t* (241) = 18.5, p < 0.001 (two tailed). Responses to an open-ended question about perceived causes of BPD were consistent with the measure; while the most common response related to genetics, many respondents referenced a combination of genetic and environmental risk factors.

### Aim 1: Coping

Participants who coped using active efforts and social support were less likely to cope using self-blame and denial. Higher use of active/social support coping was correlated with higher dispositional optimism, no endorsement of current depression, and less severe perceptions of BPD. Higher self-blame/denial coping was correlated with lower dispositional optimism, endorsement of current depression or mania, and more severe perceptions of BPD. Those participants who endorsed a genetic etiology were more likely to use active efforts/social support to cope.

A correlation matrix of key variables is presented in Table 3.

**Table 3:** Correlation of key variables

**Item**	**1**	**2**	**3**	**4**	**5**	**6**	**7**	**8**	**9**	**10**
1. Adaptation	-	-.50**	.57**	.56**	-.48**	.02	.13	.24**	.26**	.15*
2. Self-Blame/denial coping		-	-.20**	-.37**	.51**	-.04	-.22**	-.19**	-.13	-.15*
3. Active/social support coping			-	.39**	-.28**	.05	.01	.28**	.28**	.17*
4. Dispositional optimism (LOT)				-	-.46**	-.06	.08	.47**	.07	.13*
5. Self-assessed illness severity (Brief IPQ)					-	.03	-.27**	-.27**	.06	-.17*
6. Parent perception of risk for mood disorder in children						-	.02	.05	.13	.31**
7. Currently manic (self report)							-	-.05	-.05	.12
8. Currently depressed (self report)								-	.26**	.18**
9. Confidence in diagnosis									-	.27**
10. Perceived etiology measure: genetic/familial component										-

**Correlation is significant at the 0.01 level (2-tailed).

*Correlation is significant at the 0.05 level (2-tailed).

### Aim 2: Adaptation

Parents with BPD had a mean adaptation score of 2.6 (SD = 1.2), lower than scores found in eight studies of adaptation to other chronic illnesses, where it ranged from 2.7-4.2 (SD range 0.6-1.2) [9]. Multiple linear regression was used to assess the contributions of perceived illness severity, coping, dispositional optimism, depression, and participant demographics to variation in adaptation. The final model showed that active/social support coping (β = 0.4, *p* < 0.001) and dispositional optimism (β = 0.3, *p* < 0.001) were significantly associated with adaptation, and self-blame/denial coping (β = −0.3, *p* < 0.001) exhibited a negative association; together the variables explained 55.2% of the variance (F = 73.2, p < 0.001). The two items measuring participants’ perceptions of their current mania and depression were not significant in the multivariate analysis.

Self-assessed illness severity (Brief IPQ) was not significant in the final regression model. Given the strong correlation between the Brief IPQ score and adaptation, we evaluated for a mediating effect of coping on the relationship between illness severity and adaptation (see Table 4) using a series of regression analyses. The results of the mediation analysis support a mediation role for active/social support coping and self-blame/denial coping on the relationship between illness severity and adaptation.

**Table 4:** Results of mediation analyses

	**Beta**		**p-value**
Illness severity (Brief IPQ) regressed on adaptation	−0.48		p < 0.001
COPE domain regressed on adaptation	Cope 1*	Cope 2^	p < 0.001
	0.57	−0.50	
Brief IPQ and COPE domain regressed on adaptation	Cope 1*	Cope 2^	p < 0.001
	−0.35	−0.30	

*Cope domain 1 = active/social support.

^Cope domain 2 = self-blame/denial.

### Aims 3 and 4: Perceived risk to children

**Figure 1:**
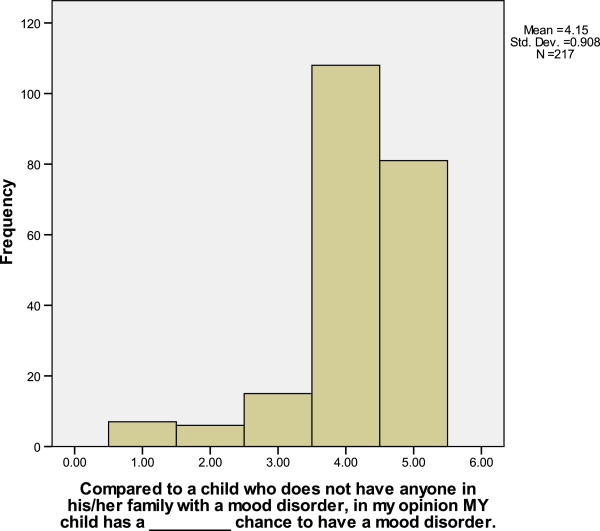
Parents’ perceived higher risk to their children compared to the population risk. 1.00 = Much smaller; 2.00 = Somewhat smaller; 3.00 = Equal; 4.00 = Somewhat greater; 5.00 = Much greater.

Eighty-seven percent of participants endorsed a “somewhat greater” or “much greater” risk for mood disorders in one’s child(ren) than someone without a family history—see Figure 1. The risk for a mood disorder in the participants’ offspring is expected to be greater than population risk, especially given the strong family histories reported (data not included). Higher perceived risk was correlated with endorsing a genetic etiology (rho = 0.31, *p* < 0.001). No confounders were significantly correlated with perceived risk, including child’s age, sex of child, birth order of child, confidence in diagnosis, and endorsement of mania or depression. In addition, participants’ dispositional optimism, perceived illness severity, coping and adaptation were not significantly correlated with perceived risk.

Parents with more than one child were asked which child they worried about the most. We also asked an open-ended question: “Why do you worry about this child the most?” The analyses of these responses revealed four themes related to increased parental concerns:

•Similarities of the child to the affected parent (i.e., in personality and behavior);

•Concerning mood states in the child (e.g., low mood, mood swings, anxiety, and attention deficit hyperactivity disorder);

•Adverse personality traits in the child (e.g., hypersensitive/emotional, angry, and poor social skills); and

•Exposure to parent’s symptoms or poor home environment resulting from parent’s illness, perceived as particularly burdensome for this child.

## Discussion

Health care providers have opportunities to improve psychological well-being for adults with BPD. Illness perceptions and coping are potential targets for non-pharmacological interventions aimed to improving overall well-being, even in individuals with BPD whose symptoms are well controlled. We identified aspects of coping that facilitate, and aspects that hinder, adaptation. Overall, respondents were only moderately well-adapted.

Consistent with our results, coping is typically conceptualized as comprising two domains—an active, positive domain and an avoidance-oriented domain. Using the BRIEF Cope measure, Meyer and colleagues [28] described similar domains in a small hospitalized population of predominantly schizophrenic patients. The relative benefits of active coping and disadvantages of avoidance coping on psychological well-being have been described in many disease populations [10]. We found that coping mediates the effect of appraisal of illness severity on adaption. In the predominantly schizophrenic population, Meyer and colleagues identified a mediating effect of measured illness severity on “adaptive” (active/support-seeking) coping, but not on “maladaptive” coping [28].

Dispositional optimism predicted coping and adaptation. A number of studies have reported a positive relationship between dispositional optimism and the use of adaptive coping strategies [30,31]. Similar to other studies [30,32] increased dispositional optimism is negatively associated with depressive symptoms, though measures of dispositional optimism are intended to measure a stable trait that is independent of mood state. Given the importance of dispositional optimism in adaptation among this sample—regardless of whether it is a stable trait or one that is influenced by the underlying pathophysiology/symptomatology of BPD—it is important to take into consideration when targeting patient interventions.

This study informs the understanding of the relationship between parents’ own illness perceptions and adaptation, and how they perceive their children’s risk of developing mood disorders. These novel findings have important implications for a holistic treatment approach in an era of increased knowledge about the etiology and pathophysiology of bipolar disorder. Appraisals of illness severity, current mood state, coping, and adaptation were not correlated with perceived risk to children, suggesting that regardless of the state of management of their own BPD, they remain aware of the risk to their children. We may have selected for a sample of parents keenly concerned about risk to their children. Among them we found little evidence of minimizing or denying risk. Data from open-ended questions suggest that parents appraise risk based on reasonable characteristics (i.e., child’s mood and personality traits, similarity to affected parent, and adverse home environment based on parent’s illness). Future research may contribute to a more complete understanding of predictors of risk perception.

Limitations of this study include the use of a sample with self-reported bipolar disorder and a likelihood of selection bias for participants functioning well enough to complete the survey and interested in the research question. Similar to most survey populations, the sample lacks racial diversity and includes more females than males; data is not available on education or economic status. These biases limit generalisability to the population of parents with BPD. The survey did not include a characterization of illness course, which may have moderated participants’ responses. A response rate is not available because the total number of individuals who had access to the web link but chose not to participate is unknown. Keeping in mind these limitations, our findings represent an important start to understanding the experiences of adults with BPD and their impact on their psychological well-being.

## Conclusions

This study suggests the need for intervention studies to evaluate the effects of enhancing active/social support coping and minimizing self-blame/denial coping on adaptation. Faced with limited health care resources, interventions might best be targeted towards those with less optimism or greater depressive symptoms. Further studies of the role of illness appraisals in adaption to BPD are needed; such studies might explore whether Coping Effectiveness Training (CET), an evidence-based intervention aimed at helping participants identify areas of control and maximize active coping in those areas [33], might enhance the relationship between appraisals and coping.

The positive relationship between endorsing a genetic/familial etiology and both active/social coping and risk perception reinforces the potential utility of genetic counseling in this population. Genetic counseling that refines clients’ understanding of BPD etiology and related family risk may help patients manage uncertainty [34] and worry, and facilitate coping and adaptation.

## Competing interests

The authors declare that they have no competing interests.

## Authors’ contributions

HLP and BBB designed the study with input from DLR. HLP collected the data and conducted the statistical analysis. All authors read and approved the manuscript.

## Pre-publication history

The pre-publication history for this paper can be accessed here:

http://www.biomedcentral.com/1471-244X/13/327/prepub

## Supplementary Material

Additional file 1 Perceived Etiology of BPD Measure. Click here for file
