# Supplementary material for: Adaptation to bipolar disorder and perceived risk to children: a survey of parents with bipolar disorder
Source: BMC Psychiatry. 2013 Dec 2;13:327. doi: 10.1186/1471-244X-13-327 (PMC3879194; doi:10.1186/1471-244X-13-327)
Supplement: Additional file 1 — Perceived Etiology of BPD Measure. [file 1471-244X-13-327-S1.doc]

**Additional file 1**. Perceived Etiology of BPD Measure

| “**Your thoughts about bipolar disorder and why it happens in families”** | | | |
| --- | --- | --- | --- |
| Scale 1-5, “strongly disagree” to “strongly agree” | | | |
|  | *Component 1:*  **Genetic/Familial** Chronbach’s alpha 0.7 | *Component 2 :*  **Attributes/Environment**  Chronbach’s alpha 0.8 | % endorsing “agree**”** or “strongly agree**”** |
| Bipolar disorder tends to run (or reappear) in families. | 0.9 |  | 80.8 |
| Bipolar disorder is likely to happen to more than one person in a family because relatives tend to share genetic factors. | 0.9 |  | 84.3 |
| Bipolar disorder is likely to happen to more than one person in a family because relatives tend to share the same home environment. |  | 0.9 | 26.3 |
| Bipolar disorder is likely to happen to more than one person in a family because relatives tend to share habits. |  | 0.9 | 19.7 |
| Bipolar disorder is likely to happen to more than one person in a family because relatives tend to share personality traits. |  | 0.8 | 41.6 |
